# Simultaneous Occurrence of Transient Complete Heart Block With Acute Lateral Medullary Infarct

**DOI:** 10.7759/cureus.60796

**Published:** 2024-05-21

**Authors:** Vishwa Pakeerathan, Abdul Rahman Mohammed, Rahil Moriswala

**Affiliations:** 1 Medicine, Princess Alexandra Hospital, Brisbane, AUS; 2 Cardiology, Townsville University Hospital, Townsville, AUS

**Keywords:** second-degree heart block, focal neurological deficits, thunder clap headache, cardiac arrythmia, lateral medullary syndrome (wallenberg syndrome), acute ischaemic stroke, posterior circulation stroke, transient third-degree av block, atrioventricular heart block, lateral medullary infarction

## Abstract

Lateral Medullary Syndrome (LMS) poses clinical challenges, often resulting from ischemic events in the medulla oblongata. We present a unique case of LMS in a woman in her 60s with a complex medical history. Alongside neurological deficits, she experienced a transient high-grade atrioventricular block (HgAVB), a rare cardiac manifestation linked to LMS. Given the rarity of simultaneous transient HgAVB with acute lateral medullary infarct, only a handful of case reports documenting similar findings are available in the existing literature. More research and case reporting are needed to better our understanding of this area. The patient's condition, marked by a sudden onset of severe headache and left-sided weakness, revealed an acute infarct in the medulla territory. Notably, her HgAVB spontaneously resolved after 72 hours. This case emphasises the crucial need for extended cardiac surveillance in LMS patients due to their susceptibility to life-threatening arrhythmias. The intricate interplay between the brainstem and cardiovascular system highlights autonomic dysregulation as a potential mechanism for cardiac abnormalities. The report advocates for a holistic approach to managing LMS cases, stressing interdisciplinary collaboration for timely diagnosis and intervention, ultimately improving patient outcomes and reducing the risk of fatal arrhythmias.

## Introduction

Lateral Medullary Syndrome (LMS) stands as a clinically intricate condition, often arising from ischemic events in the medulla oblongata [1]. While not a prevalent health problem in the general population, the challenges posed by LMS are paramount due to its potential life-threatening complications. The unique case presented in this manuscript sheds light on the interplay between neurological deficits and cardiac manifestations in LMS, emphasising the need for extended cardiac surveillance in affected individuals [1,2]. The simultaneous occurrence of a transient high-grade atrioventricular block (HgAVB) with an acute lateral medullary infarct adds a novel dimension to our understanding of autonomic dysregulation in LMS patients. Given the infrequency of concurrent transient high-grade atrioventricular block (HgAVB) with acute lateral medullary infarct (LMI), only a limited number of case reports delineating analogous presentations are documented in the current literature [1-4]. Unlike more common health issues, LMS requires a nuanced and interdisciplinary approach for timely diagnosis and intervention. This case report delivers a clear message regarding the importance of holistic monitoring and collaboration between neurology and cardiology in managing LMS cases.

## Case presentation

An Indigenous woman in her 60s presented to our Emergency Department with a severe thunderclap headache upon waking, associated with pre-syncope, dizziness, and left-sided facial weakness. Her background included poorly controlled type 2 diabetes mellitus (HbA1c 9.2%), hypertension, dyslipidaemia, and emphysema. She remained an active smoker of 25 pack years. Specifically, there is no personal or familial history of cardiac conduction disease.

**Figure 1:**
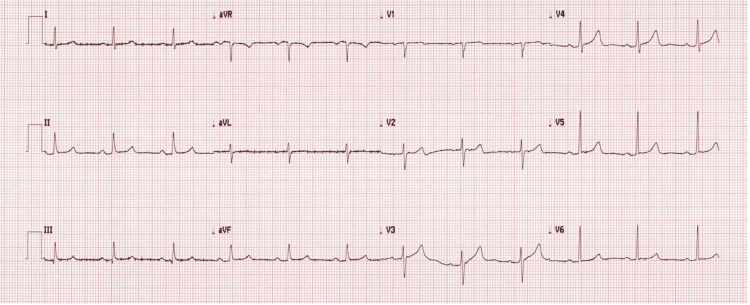
The patient's electrocardiogram on admission The electrocardiogram demonstrates normal sinus rhythm.

**Figure 2:**
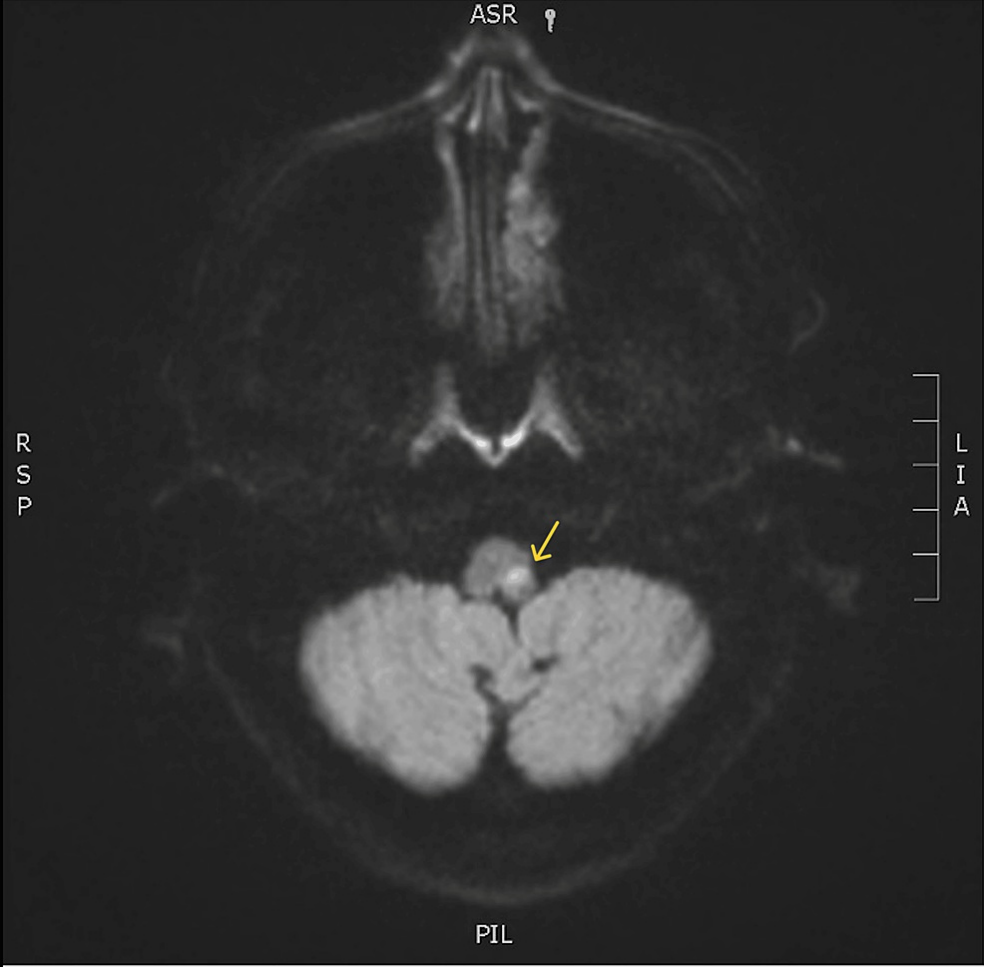
MRI brain image The image reveals a small area of true diffusion restriction with an associated T2 high signal in the left posterolateral medulla, consistent with acute infarct in the left Posterior Inferior Cerebellar Artery (PICA) territory.

Upon examination, her blood pressure was 202/87 mmHg with a regular heart rate of 85 beats/min. The neurological examination revealed left-sided lower facial weakness, decreased left-sided grip strength, and right-sided altered temperature sensation. Additionally, bulbar signs of dysarthria and rightward tongue deviation were noted. Her gait was ataxic, and she had a leftward lean. The electrocardiogram noted normal sinus rhythm (Figure 1). Her CT brain and cerebrospinal fluid (CSF) analysis had not been suggestive of a subarachnoid haemorrhage or an inflammatory process. MRI brain had shown a small area of true diffusion restriction and associated high-T2 signal in the left posterolateral medulla, consistent with acute infarct in the left posterior inferior cerebellar artery (PICA) territory (Figure 2).

**Figure 3:**
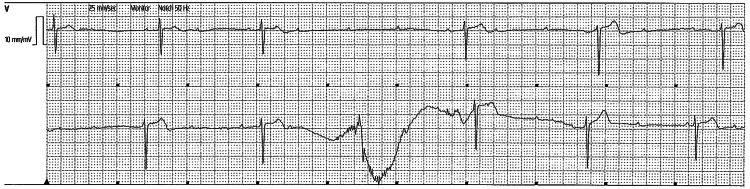
The patient's telemetry (Day 1 of admission) The telemetry results were suggestive of periods of 3rd-degree heart block.

**Figure 4:**
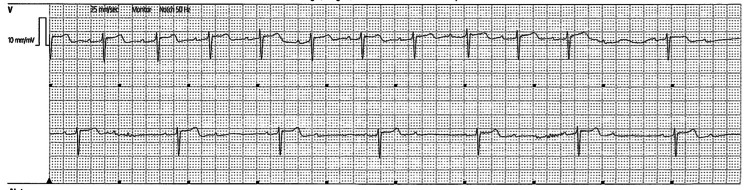
The patient's telemetry (Day 2 of admission) The telemetry result was suggestive of a high-grade atrioventricular (AV) block.

She had continued to report pre-syncopal symptoms at rest. Telemetry revealed findings of complete heart block and 2:1 HgAVB with no circadian pattern. In total, there had been five distinct episodes of complete atrioventricular (AV) blockade with more frequent 2:1 AV blocks (Figures 3, 4) while awake. Her renal function was normal, with a normal electrolyte profile. She had not been on any rate-controlling agents. A transthoracic echocardiogram showed preserved left and right ventricular function with normal valvular structures.

**Figure 5:**
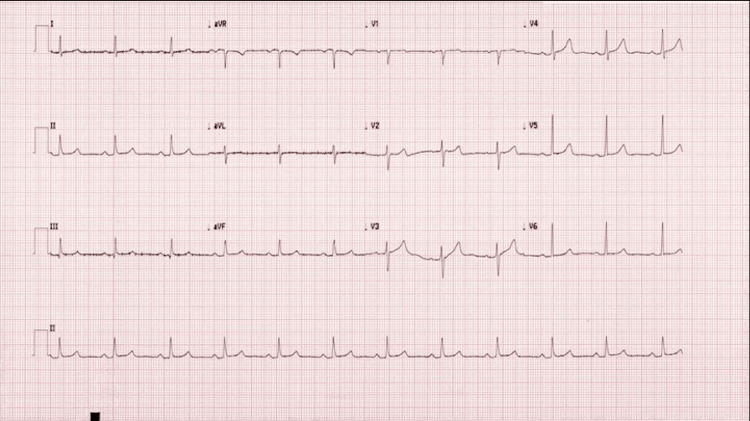
12-lead electrocardiogram 60 days after last known bradyarrhythmia suggestive of 1:1 conduction

The symptomatic bradyarrhythmia self-resolved 72 hours into her admission and there was no evidence of conduction disease on the follow-up review (Figure 5). She completed neurological rehabilitation and has been discharged to the community.

Outcome and follow-up

No events of palpitations, pre-syncope or syncope have been reported by the patient on subsequent follow-up of the patient four months later. She continues outpatient rehabilitation in her rural community.

## Discussion

This is a rare case that demonstrates a lateral medulla infarct with simultaneous transient high-grade AV block. Cerebral infarction, in this case, was secondary to occlusion of the PICA, which supplies the lateral medulla. Affected structures can include the solitary tract nucleus (STN), which collects afferent impulses from the carotid body and sinoatrial node. The dorsal motor nucleus of the vagus nerve (DMV) may also be affected, and it plays a role in efferent responses to heart function [1]. A theorised mechanism to explain the phenomenon of AV blockade is direct autonomic inhibition of the DMV and STN [1]. Medullary infarcts may disrupt central sympathetic inhibition of the STN, leading to increased vagal tone, which manifests as bradycardia and, at times, asystole [2].

A case reported by Alloush et al. involved a lateral medullary infarct with recurrent syncope. Cardiac monitoring identified sinus arrest, which responded to permanent cardiac pacing, supporting this suggested cardioinhibitory mechanism [3]. In contrast, our case and others demonstrated a transient nature of disease, which may relate to the overall resolution of neurological deficits [2,4].

Learning points and take-home messages

The learnings gathered from this study can be summarised as follows: (1) Increased cardiac risk: medullary lesions elevate the risk of autonomic cardiac injury, necessitating heightened clinical awareness; (2) Holistic monitoring: the presented lateral medullary syndrome case, coupled with transient AV blockade, underscores the importance of extended cardiac monitoring; (3) Interdisciplinary approach: managing such cases requires a holistic approach, emphasising interdisciplinary collaboration between neurology and cardiology; (4) Autonomic dysregulation: recognising the intricate interplay between the brainstem and cardiovascular system is crucial for understanding and addressing cardiac abnormalities in medullary syndrome; (5) Enhanced patient outcomes: meticulous monitoring and timely intervention can improve patient outcomes, mitigating the risk of potentially fatal arrhythmias in this vulnerable population.

## Conclusions

This case report illustrates a rare and intriguing presentation of simultaneous transient complete heart block with acute lateral medullary infarct (LMI). The occurrence of transient high-grade atrioventricular block (HgAVB) alongside LMI underscores the complexity of this clinical entity and highlights the need for heightened clinical vigilance in patients with neurological deficits. While the exact mechanism linking LMI to cardiac conduction abnormalities remains speculative, our case adds to the limited body of literature on this topic, emphasising the necessity for further research and case reporting to elucidate underlying pathophysiological mechanisms and optimal management strategies. The successful resolution of HgAVB in our patient underscores the potential for spontaneous recovery but also underscores the importance of extended cardiac surveillance in LMI patients due to their susceptibility to life-threatening arrhythmias. This case underscores the importance of interdisciplinary collaboration between neurology and cardiology in the holistic management of patients with LMI, ultimately aiming to improve patient outcomes and reduce the risk of fatal arrhythmias.
